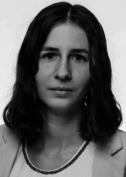# Current Situation of the Challenging Scale‐Up Development of Hydroxymethylfurfural Production

**DOI:** 10.1002/cssc.202000581

**Published:** 2020-06-04

**Authors:** Catherine Thoma, Johannes Konnerth, Wilfried Sailer‐Kronlachner, Pia Solt, Thomas Rosenau, Hendrikus W. G. van Herwijnen

**Affiliations:** ^1^ Area Wood Materials Technologies Wood K Plus—Kompetenzzentrum Holz GmbH Altenberger Str. 69 4040 Linz Austria; ^2^ Institute of Wood Technology and Renewable Materials Department of Material Science and Process Engineering BOKU- University of Natural Resources and Life Sciences Konrad Lorenz Str. 24 3430 Tulln Austria; ^3^ Institute of Chemistry of Renewable Resources Department of Chemistry BOKU University of Natural Resources and Life Sciences Muthgasse 18 1190 Vienna Austria

**Keywords:** biomass, hydroxymethylfurfural, industrial production, process development, sustainability

## Abstract

Hydroxymethylfurfural (HMF) is a high‐value platform chemical derived from renewable resources. In recent years, considerable efforts have been made to produce HMF also at industrial scale, which still faces some challenges regarding yield as well as sustainable and economic process designs. This critical Review evaluates the industrial process development of sustainable biomass conversion to HMF. Qualitative and quantitative guidelines are defined for the technological assessment of the processes described in patent literature. The formation of side products, difficulties in the separation and purification of HMF as well as catalyst regeneration were identified as major challenges in the HMF production. A first small‐scale, commercial HMF production plant with a capacity of 300 t_HMF_ per year has been operating in Switzerland since 2014.

## Graphical abstract

**Not easy peasy**: 5‐Hydroxymethylfurfural (HMF) is a bio‐derived platform chemical. The Review provides a critical assessment of developed processes for its synthesis and their applicability for scale‐up. Despite one operating HMF production plant, major chemical and technological challenges, such as the formation of side products, difficult separation and purification of HMF, and catalyst recycling, as well as challenging economics remain to be solved before large‐scale production is widely performed.

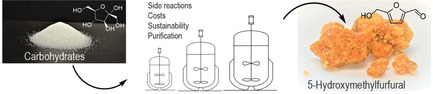

## 1 Introduction

The limited amount of fossil resources and rising environmental concerns related to CO_2_ emissions have drawn public and scientific attention to more sustainable ways of chemical production. For a sustainable development, the use of hazardous materials and fossil resources should be minimized or avoided, whereas the use of renewable resources should be enhanced.^1^ Hydroxymethylfurfural (HMF) is a promising molecule derived from renewable resources. It is a key intermediate between biomass and biochemicals and has the potential to replace a range of conventionally produced building blocks. It has been referred to as “sleeping giant”^2^ due to the anticipated enormous market potential of HMF and its derivatives. The US Department of Energy listed 2,5‐furandicarboxylic acid (FDCA), a HMF derivative, as one of twelve top value‐added chemicals in 2004. It is a promising starting block for polyethylene 2,5‐furandicarboxylate (PEF) synthesis, which is a bio‐derived alternative to polyethylene terephthalate (PET).^3^ The production of bioplastics from bio‐based chemicals has come into the focus of several industries. PEF is a promising bioplastic with excellent gas‐barrier performance, recyclability, and extended mechanical properties.^4^

**Figure 1:**
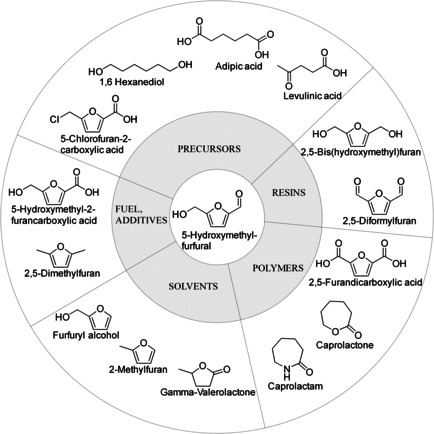
Value‐added biobased chemicals derived from HMF.

A short overview of chemical compounds derived from HMF and their potential application fields is given in Figure 1.

HMF is a key intermediate for valuable chemicals out of C_6_‐carbohydrate (hexose) building blocks, for example, levulinic acid, 1,6‐hexanediol, or adipic acid. Its derivatives, for example, 2,5‐bis(hydroxymethyl)furan^5^ or 2,5‐diformylfuran,^6^ are promising cross‐linkers in the resin production. These biobased resins are capable replacements of currently used, fossil‐based adhesives in various industries, such as foundry or wood industries. In addition, the potential of HMF derivatives as solvents^7^ and fuels^8^ has been reported. 2,5‐Dimethylfuran is an alternative biofuel, with a volumetric energy density comparable to gasoline. It is insoluble in water, stable in storage as it will not be contaminated through water absorption from atmosphere, and has a high research octane number.^9^

The commercial production of chemicals concerns economic, environmental, and industrial aspects. For a sustainable development the chemical product should be derived from biobased chemicals such as HMF; in addition, the negative environmental impact of the processing and manufacture must be minimized. A sustainable chemical product must satisfy both sides, the producer and the consumer. It has to be a commercial success for the producer and still be affordable for the end‐user.^10^

Various reviews were published on the laboratory‐scale synthesis of HMF. The reviews mainly focused on solvents^4^, ^11^ and catalytic systems.^12^ Reviews on the used feedstocks,^13^ biological properties, and its synthesis and applications^14^ can be found in the literature as well. A review on the chemistry of HMF, process technologies, and its application as platform chemical was published by van Putten et al.^15^ in 2013. Since then, the implementation of industrial‐scale HMF production processes has gained much more attention, and an increasing number of HMF production methods have been patented in the last couple of years. A review with a more holistic point of view, which connects the work from academia and industry, is still needed.^16^

The aim of this Review is to bridge this gap and identify the biggest challenges researchers face in the development of HMF production methods and to critically assess the developed process technologies and the applicability for industrial systems. It also provides an overview of the main improvements in process development, especially with regard to green processing.

### 1.1 Principles of monosaccharide dehydration to HMF

**Figure 2:**
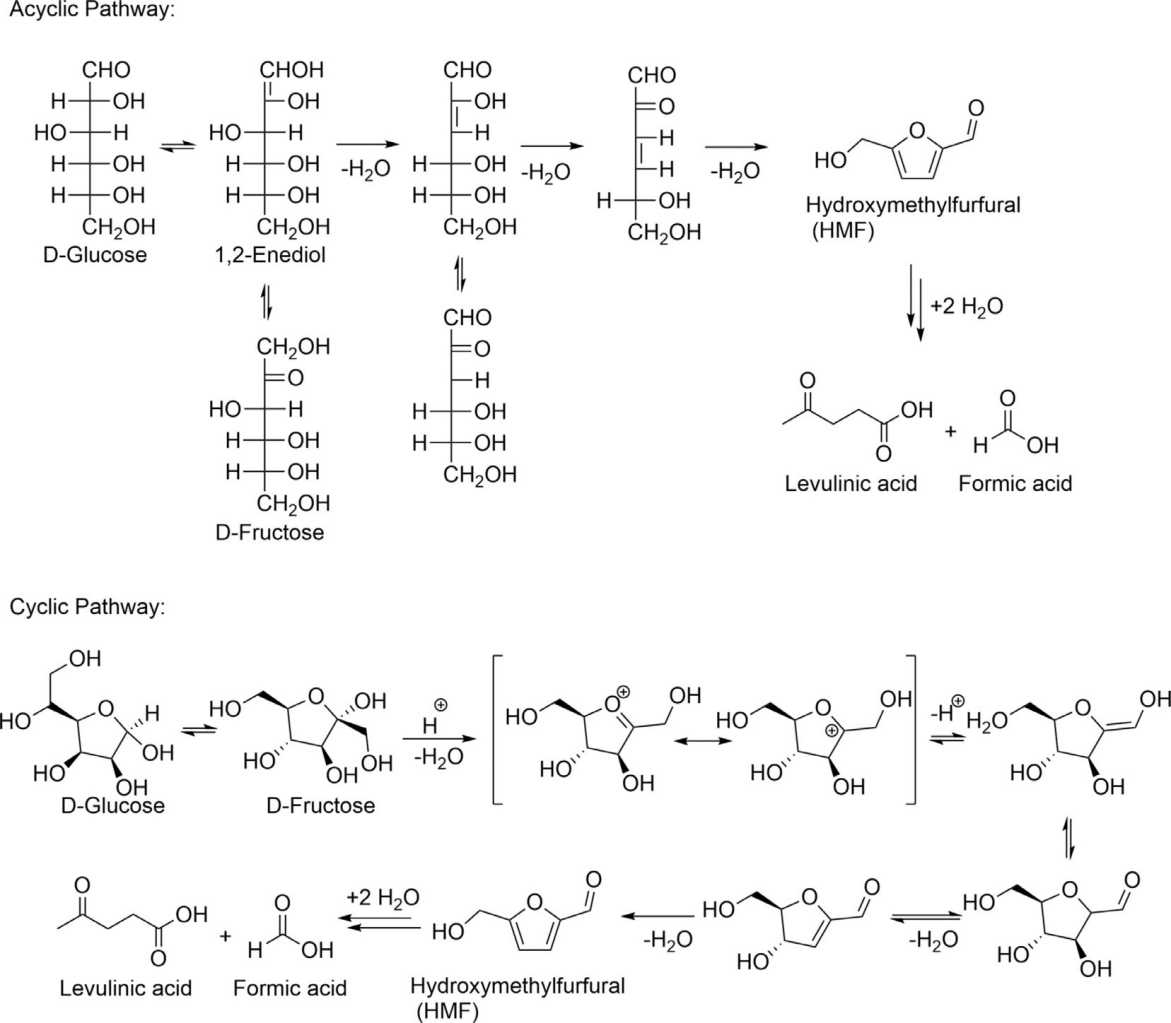
Proposed mechanisms of HMF formation from hexoses.^18^

HMF combines the functionalities of furfural and furfuryl alcohol. Characteristics of HMF are its hydroxyl and aldehyde group as well as the furan ring, as can be seen in Figure 2.

The thermal, acid‐catalyzed dehydration of hexoses, for example, glucose or fructose, results in the formation of HMF. Several kinetic studies on the HMF formation from various biomass feedstock were summarized by van Putten et al.^15^ Kinetic studies^17^ can not only be used to get insight into the mechanisms of the HMF formation on molecular level but serve also as input for the development of optimum reactor configurations and process conditions.

Several mechanisms have been proposed for the formation of HMF from hexoses.^18^ The direct formation of HMF by acid‐catalyzed dehydration is generally described as the removal of three water molecules from the sugar molecule. Depending on the structure of the formed intermediates, the proposed mechanisms can be divided into cyclic and acyclic routes.^18^ There have also been ^13^C isotopic labelling studies for fructose dehydration,^19^ but no definite proof for either of the mechanistic routes has yet been published for the HMF case. However, for the structurally closely related system of hexeneuronic acid (4‐deoxy‐β‐l‐*threo*‐hex‐4‐enopyranosiduronic acid), leading to 5‐formyl‐2‐furoic acid by triple dehydration, the occurrence of both acyclic and cyclic intermediates has been demonstrated by a combination of ^13^C isotopic labelling and NMR spectroscopy.^20^ In addition, solvent effects make the comparison of kinetic parameters for dehydration reactions in biphasic water–organic solvent mixtures and monophasic systems difficult.17c

### 1.2 Challenges of HMF synthesis: Side reactions and isolation

HMF can be derived from hexoses, preferably from hexoketose d‐fructose. The formation of HMF from fructose often entails some side reactions, such as isomerization, fragmentation, and condensate formation.^18^ The HMF yield obtained from d‐fructose is higher than from d‐glucose under the same reaction conditions. Since d‐glucose is cheaper, several studies also focused on the HMF synthesis from glucose.^15^ Isomerization of glucose to fructose seems to be a required step in the synthesis of HMF from glucose, making an efficient isomerization catalyst necessary. Since the glucose–fructose isomerization is best base‐catalyzed and the following dehydration of fructose is acid‐catalyzed, this has spurred some research on the catalytic systems, especially on bifunctional catalysts.12a

HMF reacts in aqueous mixtures with two water molecules in a rehydration reaction, forming levulinic acid and formic acid (Figure 2), sometimes referred to an Achmatowicz‐type process.^21^ This degradation decreases the overall HMF yield and makes expensive purification processes necessary. The rehydration of HMF is suppressed in non‐aqueous systems. The dehydration reaction is accompanied by condensation reactions, which form a black tarry by‐product consisting of complex furanic oligomers called humins.^22^ They have recently been shown to consist of quinoid–furanoid ladder‐type oligomers rather than of linear polymers as previously assumed.^23^ Their extremely high extinction coefficients account for their black appearance. From an economical and technological perspective, the formation of humins is highly undesired. In general, it lowers the efficiency of the dehydration process, renders purification and decoloration difficult, and decreases catalyst efficiency. Recent publications focused on finding new ways for humins valorization to turn those drawbacks into an advantage.^24^

The presence of condensation products causes major problems, especially for HMF separation and purification. The recovery of HMF is associated with difficulties due to its thermal lability under long‐term heating in both alkaline and acidic conditions. Thus, separation of HMF from the reaction mixture, for example, by distillation, is challenging. In a recent publication, Gomes et al.^25^ described the enhanced thermal stability of HMF during synthesis in biphasic systems and distillation in the presence of sodium dithionite. Without the addition of sodium dithionite, the formation of degradation products, mainly tarry carbonaceous material was formed. Furthermore, HMF is difficult to store due to its relative instability and sensitivity towards acids, alkali, and oxygen even under mild conditions.^26^ Galkin et al.^27^ showed that during two weeks of storage of a HMF oil with 97–99 % purity decomposition took place, leading to the formation of dimers and larger oligomers.

The separation and purification of HMF is one of the most important challenges in the scale‐up of HMF production.

## 2 Process Assessment Criteria

**Figure 3:**
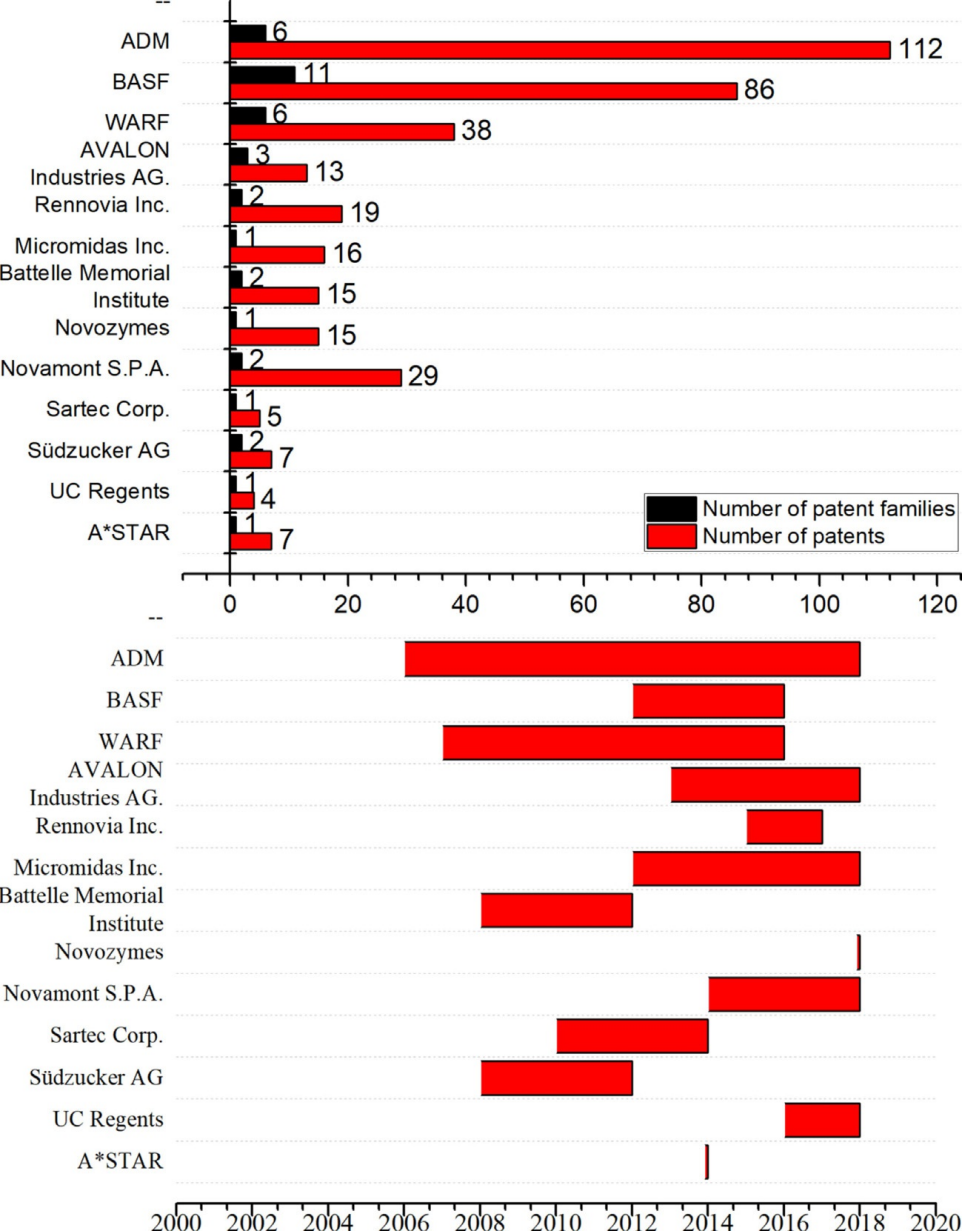
Number of patents in HMF technology of top assignees (top), timeline of patent assignments in HMF technology from 2006 to present (bottom).

Based on the number of patent applications related to HMF production there has been a continuous growth of interest in this topic as can be seen in Figure 3. Several adjustments to existing HMF production methods have been made to improve the chemical and economic efficiency of potential HMF production processes. The related research can roughly be divided into the main fields given in Table 1.

**Table 1:** Research topics and defined process assessment criteria.

Process assessment criteria	Research topics
operational aspects	operating mode
	reactor design
solvent system	single‐phase systems
	biphasic systems
catalytic system	salts
	acid cation exchange resin
	metal halides
	mineral acids
feedstock selection + conversion	isomerase enzymes
	partial conversion endpoint
	production of HMF from by‐products

Methods have been developed and adjusted starting from basic operational variations, for example, different operation modes and reactor designs for a better control of temperature and reaction time, to the testing of single‐phase and biphasic reaction mixtures and different catalytic systems to reduce the side reactions. In terms of feedstock selection, the use of isomerization enzymes has been proposed to increase the HMF yield from saccharides. Several production methods are based on setting a partial conversion endpoint to limit the formation of follow‐up products and increase the efficiency of HMF production. The production of HMF from agricultural side products was developed to make the process more economic. The production methods described in patent literature each have its benefits and drawbacks, the most critical factor being the HMF yield.

The process assessment of the upscaled HMF production methods is limited due to the lack of data provided in the patents. The product yield and the reaction mass efficiency (RME) are the parameters that describe the efficiency of a process and are thus needed for a quantitative process assessment. Yet neither product yield nor RME consider byproducts, wastes, solvents, catalysts, or energy issues. A qualitative analysis of the used catalyst, solvent systems, and feedstock is, however, still possible and can point out options and directions for future process developments.

One strategy for the development of greener processes is the appropriate selection of the solvent.^10^ With regard to the “greenness” of a process, solvents are usually an environmental concern due to the typically large quantities used. The Innovative Medicines Initiative (IMI)‐Chem21^28^ published a comparative survey of different solvents resulting in selection guides. The Chem21 classification of the solvents used in the described processes is included in Table 2. As can be seen, the most sustainable solvents for HMF production are alcohols, water, and methyl isobutyl ketone (MIBK).

**Table 2:** Comparison of HMF processes and relevant parameters of early HMF production methods (before 2006).

Company/Research center	Reactor type	Process type	Feedstock	Catalyst	Solvent (Chem21 classification)	*T* [°C]	*t* [min]	Yield [%]	Ref.
Food Chemical and Research Laboratories Inc.	autoclave	batch	saccharose	H_2_SO_4_	butanol/water (recommended)	150	20	68.6^[a]^	32
Food Chemical and Research Laboratories Inc	glass tube	batch	fructose	H_2_SO_4_	butanol/water (recommended)	170	8	68.0	32
Dendrol Inc.	pressurized batch	batch	oak wood chips	0.6 % H_2_SO_4_	water (recommended)	285.56	1.5	8	33
Merck Co Inc,		batch	saccharose	–	water (recommended)	270	0.75	37.2	34
Merck Co Inc.	tubular reactor	batch	saccharose	0.04 % levulinic acid	water (recommended)	256		30‐31	34
Merck Co Inc.		batch	fructose	NH_4_Al(SO_4_)_2_	water (recommended)	270	0.15	58	35
Merck Co Inc.		batch	glucose	NH_4_Al(SO_4_)_2_	water (recommended)	270	0.18	50.8	35
Merck Co Inc.		batch	saccharose	NH_4_Al(SO_4_)_2_	water (recommended)	270	0.18	46	35
Merck Co Inc.		batch	saccharose	Al(SO_4_)_2_	water (recommended)	290	1.03	49.7	35
Merck Co Inc.		batch	glucose	AlCl_3_	water (recommended)	240	2.5	50.0	35
Merck Co Inc.		batch	fructose	Al(SO_4_)_3_×18 H_2_O	water (recommended)	271	0.18	50	35
Atlas Ind.	glass tube	batch	sorbose	H_2_SO_4_	methoxyethanol, water	180	3	52.1	36
Atlas Ind.	glass tube	batch	sorbose	H_2_SO_4_	bis(chloroethyl) ether	180	1.8	52.2	36
Atlas Ind.	glass tube	batch	sorbose	H_2_SO_4_	MIBK+water (recommended)	180	3	52.5	36
Atlas Ind.	glass tube	batch	sorbose	HCl	mesityloxide	180	2	56.4	36
Atlas Ind.	glass tube	batch	glucose	AlCl_3_	dioxane, water (hazardous)	210	3	41.6	36
Atlas Ind.	glass tube	batch	glucose	AlCl_3_	dimethyldioxane	210	2.5	40.9	36
Atlas Ind.	glass tube	batch	saccharose	CrCl_3_ +HCl	dioxane, water (hazardous)	120	4	40.2	36
Atlas Ind.	glass tube	batch	saccharose	AlCl_3_ +HCl	dioxane, water (hazardous)	150	23	40.2	36
Atlas Ind.	glass tube	batch	sorbose	HCl	triethylene glycol	180	3	66.9	36
Roquette Freres		batch	fructose	cation resin: Lewatit SPC108	MIBK/water (recommended)	85	240	89^[b]^	37
Roquette Freres		continuous	fructose	cation resin: Lewatit SPC108	DMSO (problematic)	76	6000	97	38
Südzucker AG.		batch	fructose	oxalic acid	water (recommended)	135–142	130	33.6	44
Südzucker AG.		batch	inulin	sulfuric acid	water (recommended)	140	120	13	44

[a] At 86% conversion. [b] At 21 % reaction mass efficiency.

Several reviews11b, 12a, 14, 15 were published on the catalytic systems used in HMF synthesis. For a sustainable process, high selectivity of the catalyst towards HMF generation is preferred. Up to 93 % HMF yields were obtained using ionic liquids (ILs) and acidic ion‐exchange resins.

Menegazzo et al.^13^ recently summarized the publications on the direct synthesis of HMF from raw biomass, including edible biomass, non‐edible lignocellulosic biomass, and food wastes. Hexoses, for example, fructose or glucose, have been used preferably as feedstock for HMF synthesis. The HMF yield is higher when fructose is used as feedstock, but in general glucose is more easily available and cheaper. In general, lignocellulosic biomass consisting of cellulose, hemicellulose, and lignin are a promising feedstock for the conversion to HMF since cellulose and hemicellulose can be degraded into hexoses and pentoses. Even though monosaccharides are the easiest starting materials to be converted into HMF, the additional step of obtaining monosaccharides out of polysaccharides is a drawback. Still, lignocellulosic biomass as HMF feedstock is favored from an economic and sustainable point of view.11b, 15

## 3 Early Work on HMF Production Process Development (Until 2006)

In 1895, Düll and Lintner^29^ were the first to synthesize HMF from inulin using 0.5 % oxalic acid as a catalyst. In the same year, Kiermayer^30^ dehydrated fructose under pressure using 0.3 % oxalic acid solution. Given the analytical methods of that time, it is admirable that Kiermayer identified the structure of HMF almost correctly. Haworth and Wiggins^31^ modified Kiermayers process and found that an enhanced HMF yield was obtained when saccharose is dehydrated at higher temperatures of 162–167 °C without the use of an additional catalyst. The acidic substances formed in the conversion of saccharose were found to sufficiently catalyze HMF formation.

The Food Chemical and Research Laboratories Inc.^32^ reported the formation of HMF in a patent in 1956. The reactions were performed under pressure in the presence of an acidic catalyst, for example, HCl, HBr, H_3_PO_4_, H_2_SO_4_, ZnCl_2_, or AlCl_3_. An aliphatic mono‐ol, for example, butanol, was used as the reaction medium, along with saccharose and fructose as the feedstocks. The overall yield was rather moderate. At 150 °C, the conversion of saccharose gave the highest yield of 68.6 % in a butanol/water mixture after 20 min. The reaction was performed in an autoclave. In additional experiments in glass tubes, the conversion of fructose was 68.0 % at 170 °C after 8 min. Based on the experimental data, a pseudo‐first order reaction kinetics for the HMF formation was proposed in the case of low sugar concentrations.

Dentrol Inc.^33^ filed a patent in 1958 on a process for HMF production from cellulosic raw material, such as small pieces of hardwood, for example, oak wood chips. This feedstock was dispersed in 0.6 % H_2_SO_4_ and then charged in a reaction vessel at high temperature (285.6 °C) and pressure (6.9 MPa). The dehydration reaction is performed using high‐pressure steam. The liquid condensation product contains about 8 % HMF, which is about 20 % of the theoretic yield based on the cellulose charge (based on 40 % cellulose in wood). However, the low HMF yield appeared unattractive when comparing the results to those of other studies at that time.

In 1960 Merck Co. Inc.^34^ patented a continuous process for carbohydrate conversion to HMF in aqueous solution at temperatures between 250–380 °C. A HMF yield of 37 % was obtained from the conversion of saccharose at 270 °C and a reaction time of 45 s. The formation of a black, soluble tar was also reported. In 1969, an improved method was patented,^35^ in which aluminum salts were used as the catalysts. The highest obtained yield was 58 % HMF from the conversion of fructose. In this experiment, ammonium aluminum sulfate (NH_4_Al(SO_4_)_2_⋅12 H_2_O), was used as catalyst with a reaction time of 9 min at 270 °C. The efficiency of the catalyst for the conversion of sorbose and galactose was significantly lower (27.4–37.7 % HMF). It is interesting to see that many of the early contributions to HMF process developments also tested different feedstocks, for example, wood and lignocellulosics, saccharose, galactose or sorbose, whereas later work often focused on the conversion of fructose and glucose.

Atlas Chemical Industries^36^ filed a patent on the acid‐catalyzed dehydration of hexoses to HMF in 1963, such as sorbose or glucose, or hexose disaccharides, such as saccharose. The reaction medium consisted of water and an organic solvent, for example, MIBK or dioxane. Mineral acids, such as HCl or H_2_SO_4_, were used as catalysts together with salts, for example, AlCl_3_ or CrCl_3_. The separation and recovery of HMF from the reaction mixture was not covered in the patent. The reactions were performed at 150, 180, and 210 °C. The HMF yield was related to the hexose charged and hexose consumed in the process. Overall, the obtained yields were rather moderate (40–67 %). The highest HMF yield was 66.9 % (hexose charged) and 80.0 % (hexose consumed). In this example, sorbose was reacted in triethylene glycol using 0.13 % HCl as the catalyst for 3 min at 180 °C.

A patent of Roquette Freres^37^ disclosed a process for the decomposition of hexoses in a biphasic reaction mixture at temperatures between 85–90 °C. An ion‐exchange resin with a cationic functionalization was used as the solid catalyst. The highest obtained yield was 89 % HMF, produced in MIBK as organic phase at 85 °C, catalyzed by the cation‐exchange resin Lewatit SPC 1008. Contrary to earlier studies, large volumes (40 L solvent+1 kg fructose) were used in the reaction, but the conversion rate was rather low (21 %). It is important to point out that most early studies—as well as most current work—performed the dehydration to HMF in very small quantities, with limited conclusiveness to larger‐scale processes. This makes the research of Roquette Freres a good starting point for further investigations. Increasing the catalytic efficiency and improving the yield had proven to be an important area for future work. Roquette Freres^38^ also patented a counter‐current process technology for the synthesis of HMF. The sugar‐containing starting material is dissolved in a polar aprotic solvent, for example, dimethyl sulfoxide (DMSO), in the presence of a solid catalyst at temperatures between 75–80 °C. The formed HMF is then extracted to another solvent, for example, MIBK, in a continuous counter‐current setup. In one example, the ion‐exchange resin Lewatit SPC 108 was used as the catalyst with DMSO as the reaction medium at 80 °C, giving an HMF yield of 97.5 % as determined by gas chromatography. The high yield of HMF was a clear advantage of systems using DMSO, although its role in the conversion is still not completely understood and is the subject of considerable debate.^39^

**Figure 4:**
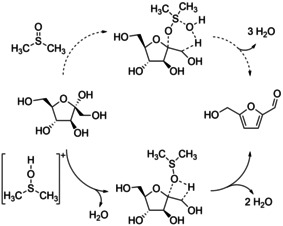
Postulated dehydration of fructose in the presence (lower pathway) and absence (upper pathway) of Brønsted acid catalyst in DMSO.

Tsilomelekis et al.^40^ analyzed the molecular structure, morphology, and generation of humins in a system that used DMSO as co‐solvent. Their analytical data supported the postulated mechanism of humin growth by van Zandvoort et al.,^41^ in which humins are formed through electrophilic attack of HMF carbonyl moieties at the α‐ or β‐position of furan rings. In their experiments, they showed that this pathway is significantly suppressed in polar aprotic co‐solvents, such as DMSO. In a previous study using frontier molecular orbital theory by Tsilomelekis et al.^42^ they found that DMSO minimizes the susceptibility to nucleophilic attack and thus rehydration and humin formation due to the reduction of the LUMO energy. They also stated that the hydrogen bond acceptor strength of DMSO is higher than that of the HMF carbonyl group. It has also been shown by Ren et al.^43^ that the isomer distribution of fructose in DMSO‐containing media is different from that in water. In DMSO, β‐d‐fructofuranose is the most stable form of fructose, whereas in water the β‐pyranose is dominant. In addition, Ren et al.^43^ postulated that in the presence of a Brønsted acids the catalytically active sulfonium species [DMSOH]^+^ is formed, which interacts with the fructofuranose isomer (see Figure 4).

In 1988, Südzucker AG^44^ patented a batch process for producing HMF in aqueous media. Oxalic acid was used as catalyst. Fructose or inulin from chicory roots were used as feedstock, the obtained product solution was purified by column chromatography. An HMF yield of 33 % could be reached from the dehydration of fructose after 130 min at 135–142 °C. When using inulin and H_2_SO_4_, an HMF yield of 13 % and 30 % fructose was obtained at 140 °C after 120 min. Humins were formed as side product and filtered off before the purification process. HMF (99 % purity) was obtained after crystallization. The yield is significantly lower than those of processes in previously described literature. The formation of humins was reported.^44^

## 4 More Recent Process Development for HMF Production (2006 to Present)

Since 2006, the number of publications and patents on HMF production methods has increased steadily. Several companies developed processes for carbohydrate conversion to HMF, and many of them patented multiple process methods and also improved their process concepts. Details on operational aspects, solvent selection, catalytic systems, and feedstock selection are discussed in this section as far as available in literature and separately for each process. For a better comparison of the specific details on the conversion to HMF, the information is summarized in Table 3. The table is structured in accordance with the text based on the company that filed a patent for the respective process concept.

**Table 3:** Comparison of HMF processes and relevant parameters of HMF production methods.

Company/Research center	Reactor type	Process type	Feedstock	Catalyst	Solvent (Chem21 classification)	*T* [°C]	*t* [min]	Conv. [%]	Yield [%]	RME [%]	Ref.
BASF SE	tank reactor	batch	fructose		ionic liquid, [EMIm][MeSO_4_]	100–160			88		45
BASF SE	tank reactor	batch	glucose	metal chloride, CrCl_3_	ionic liquid, [EMIm][MeSO_4_]	100–160			63		45
BASF SE		semi‐batch	fructose	Brønsted acid, methanesulfonic acid	ionic liquids, [EMIm][OMs]			97.6	86.5	60.7	46
BASF SE	tank reactor	batch	fructose	Brønsted acid, methanesulfonic acid	ionic liquids, [EMIm][OMs]	100–160		93	75.8		46
BASF SE	pipe reactor	continuous.	fructose	Brønsted acid, methanesulfonic acid	ionic liquids, [EMIm][OMs]	100–160		97	71.4		47, 88
BASF SE	evaporator	continuous.	carbohydrates		ionic liquid+organic solvent				22		44, 88
BASF SE	evaporator	continuous.	fructose		[BMIm]Cl+methanol+water (recommended)	200			8		47, 88
ADM SE		batch	HFCS								47
ADM SE		batch	HFCS	Amberlyst 35	NMP (hazardous)	115	300	94.1	80.6		48
ADM SE		batch	HFCS	Amberlyst 35	NMP (hazardous)	105	300	71.6	85.4		48
ADM SE		batch	HFCS	Amberlyst 35	DMAc (hazardous)	105	300	62.1	74.6		48
ADM SE		batch	HFCS	Amberlyst 35	DMF/MIBK (hazardous/recommended)	85	420	89.3			48
ADM SE	in situ distillation	batch	HFCS	Amberlyst 35	NMP (hazardous)	105	120	75.7	79.5		48
ADM SE		batch	HFCS	1.8 % H_2_SO_4_	NMP (hazardous)	160	20	80.1	12.4		48
ADM SE		batch	fructose	5 % HCl	2‐butanol +NaCl (recommended)	120	30	49			50
ADM SE		batch	fructose	5 % HCl	diglyme	100	60	43			50
ADM SE		batch	fructose	5 % HCl	dioxane (hazardous)	130	15	46			50
ADM SE		batch	fructose	10 % HCl	glyme	110	30	48			50
ADM SE		batch	fructose	5 % HCl	THF (problematic)	140	30	44			50
Micromidas Inc.	fluidized bed reactor	continuous	cellulosic feedstock	dried gaseous acid, e.g., HCl	organic solvent, e.g., dichloromethane (hazardous)	200‐250					54
WARF	glass vial	batch	fructose	H_2_SO_4_	DMAc–LiCl (0.5 %) (hazardous)	120	120		81		55
WARF	glass vial	batch	fructose	CuCl; additive: [EMIm]Cl	DMAc–LiCl (10 %) (hazardous)	120	90		83		55
WARF	glass vial	batch	fructose	H_2_SO_4_	DMAc+LiI (hazardous)	120	120		94		55
WARF	glass vial	batch	fructose	H_2_SO_4_; additive: [EMIm]Br	DMAc (hazardous)	120	60		94		55
WARF	glass vial	batch	glucose	CrCl_2_	DMAc (hazardous)	120	195		45		55
WARF	glass vial	batch	glucose	CrCl_2_; additive: NaBr	DMAc (hazardous)	100	300		81		55
WARF	glass vial	batch	glucose	CrCl_2_, additive: LiBr	DMAc (hazardous)	100	240		79		55
WARF	glass vial	batch	glucose	CrCl_2_, additive [EMIm]Br	DMAc (hazardous)	100	120		78		55
WARF	glass vial	batch	saccharose	CrCl_3_, additive: LiBr	DMAc (hazardous)	100	180		79		55
WARF	glass vial	batch	cellulose	CrCl_2_+HCl; additive [EMIm]Cl	DMAc–LiCl (hazardous)	140	120		54		55
WARF		batch	cellulose	H_2_SO_4_	aprotic polar solvents, e.g., THF (problematic)	190	120		44		57
WARF		batch	glucose	AlCl_3_+HCl	biphasic: SBP, water				62		60
WARF		batch	fructose	AlCl_3_+HCl	biphasic: GVL, water (problematic/recommended)	170	20	94	84		61
Battelle Memorial Institute	glass vial	batch	fructose	PtCl_2_	[EMIm]Cl	80	180	99	83		63a
Battelle Memorial Institute	glass vial	batch	fructose	RhCl_3_	[EMIm]Cl	80	180	99	83		63a
Battelle Memorial Institute	glass vial	batch	fructose	H_2_SO_4_	[EMIm]Cl	80	180	99	80		63a
Battelle Memorial Institute	glass vial	batch	fructose		[EMIm][MeSO_4_]	80	180	99.6	86.5		63a
Battelle Memorial Institute	glass vial	batch	glucose	CrCl_2_	[EMIm]Cl	100	180	94.4	68		63a
A*STAR		batch	fructose	HCl	isopropanol (recommended)	100	240		83	58.1	64
A*STAR		batch	fructose	HCl	ethanol (recommended)	100	240		60	42.0	64
A*STAR		batch	fructose	HCl	1‐propanol (recommended)	100	240		73	51.2	64
A*STAR		batch	fructose	HCl	1‐butanol (recommended)	100	240		68	47.6	64
A*STAR		batch	fructose	HCl	isopropanol (recommended)	120	60		82	57.40	64
Sartec Corp.	column reactor	continuous	glucose	TiO_2_+HCl	MIBK/water (10:1)	180	2		46		66
Novamont S.P.A.	glass flask	batch	saccharose	TEAB, HPWO/Si50O	water (recommended)	80–100			67	24.68	67
Novamont S.P.A.	glass flask	batch	fructose	TEAB, HPWO/Si50O	water (recommended)	80‐100			80		67
Novamont S.P.A.	glass flask	batch	fructose	Ti Si50O, TEAC	water (recommended)	80–100			93	65.10	67
Novamont S.P.A.	glass flask	batch	fructose	α‐Zr(HPO_4_)_2_+TEAB	water (recommended)	80–100			87.6	61.32	67
Novamont S.P.A.	glass flask	batch	maize syrup	10 % HPWO/Si50O+TEAB	water (recommended)	80–100			75	59.0	67
Novamont S.P.A.	glass flask	batch	fructose	Ti(HPO_4_)_2_+TEAB	water (recommended)	80–100			80	56	67
Novamont S.P.A.	glass flask	batch	inulin	30 % HPWO/Si50O+TEAB	water (recommended)	80–110			63	60.0	67
Novozymes A/S		continuous.	fructose	salts, e.g., KCl	biphasic: organic solvent/ water			98	70		68
UC Regents		batch	glucose	acid catalyst, for example, AlCl_3_	ionic liquids, e.g., [C_2_mim]Cl^[a]^				55–60		69
Südzucker AG.	TESA reactor	continuous[]s.	fructose	1–2.5 wt % mineral acids, e.g., HCl	water (recommended)	80–165		40^[b]^			70
AVALON Industries	pressure reactor	batch	lignocellulose		water (recommended)	–		–			73

[a] C2mim=1‐ethyl‐3‐methylimidazolium. [b] Set endpoint.

### 4.1 BASF SE

BASF SE (Ludwigshafen am Rhein, Germany) worked on the fundamental understanding of the influence of the operating mode on the HMF yield by comparing reactor systems with different operation modes (semi‐batch, continuously stirred tank reactor, and pipe reactor). In 2013 and 2014, BASF SE patented^45^ a two‐step HMF production method in a continuously stirred tank reactor. First, dehydration of fructose occurs at comparatively low temperatures (100–160 °C) using the IL 1‐ethyl‐3‐methylimidazolim methylsulfate ([EMIm][MeSO_4_]) as the solvent. No additional catalyst was used for the conversion of fructose; acidic conditions are obtained automatically through autoprotolysis. Then the reaction solution is evaporated and separated from the solvent in a second reactor at elevated temperatures (200 °C). In one example, the obtained HMF yield was 79 % after the first reactor and 88 % after the second. The same experiment was performed with 70 wt % glucose solution in [EMIm][MeSO_4_] and CrCl_3_ as the catalyst. An HMF yield of 63 % was obtained after the second reactor. The use of metal chlorides as effective Lewis‐acid catalysts for the synthesis of HMF from fructose or glucose has already been described in literature.12a In a recent publication, Zhou et al.17d found in laboratory experiments that the catalytic ability varies with the type of cation (FeCl_3_⋅6 H_2_O, CrCl_3_⋅6 H_2_O, AlCl_3_) since the acidity and the coordination ability with ligands are critical. Using metal chloride catalysts, the HMF yield decreased in the order H_2_O<1‐butyl‐3‐methyl imidazolium chloride ([BMIm]Cl)<DMSO. The utilization of ILs stabilized HMF in the reaction solution, which was evidenced by the mechanistic study of Zhou et al..17d They found that the sugar–metal coordination is responsible for the catalysis of [BMIm]Cl and metal halides. The separation of HMF from ILs is usually quite difficult and affords large volumes of solvents for extraction. With this in mind, the question arises if the BASF process can be optimized beyond the obtained yield by varying the solvent and metal chloride catalyst. Unfortunately, no additional information was given in the BASF patent on this aspect. More recently, BASF SE^46^ also compared the utilization of operation modes (semi‐batch, continuously stirred tank reactor, pipe reactor) for the dehydration of fructose in the IL 1‐ethyl‐3‐methylimidazolium methylsulfonate ([EMIm][OMs]) using methanesulfonic acid as the catalyst. The influence of the solvent on the HMF yield was as follows: [EMIm][OMs]>1‐ethyl‐3‐methylimidazolium chloride ([EMIm]Cl)>1‐butyl‐3‐methylimidazolium mesylat ([BMIm][OMs])>1‐ethyl‐3‐methylimidazolium tosylate ([EMIm][OTs])>1‐ethyl‐3‐methylimidazolium hydrogen sulfate ([EMIm][HSO_4_]). For these studies, a Brønsted acid with an anion corresponding to the anion of the IL was used as the catalyst. BASF SE reported the highest HMF yield of 86.5 % at 97.6 % conversion for the reaction in a semi‐batch process. Using a continuously stirred tank reactor, the HMF yield was reduced to 75.8 % at 93 % conversion. The yield of this experiment lies in the range of previously patented dehydrations in batch reactors by BASF SE. In the pipe reactor, an overall yield of 71.4 % HMF at 97 % conversion was achieved. Interestingly, the HMF yield in the continuous process was significantly lower than in the semi‐batch processes. The separation of HMF from the product solution is done by short‐path evaporation. However, a closer look to the used fructose concentration points out some problems regarding the efficiency of the reaction. Even though a high HMF yield (86.5 %) was obtained in the semi‐batch process, only a 20 wt % fructose solution was used as the feedstock. When using a more highly concentrated fructose solution of 65 %, the HMF yield dropped to only 50.9 %. A higher concentration of the feedstock would increase the efficiency and sustainability of the process since less solvent and less energy are needed for the separation of product from solvent. Even though the comparison of different operation modes shed some light on the fructose conversion to HMF, there are many other parameters that might have an influence on the result, ranging from better mixing and improved heat and mass transfer to lower residence times, lower concentrations, and smaller concentration gradients. Many of these questions remain unanswered.

Besides varying the operating mode, the utilization of different reactor designs was reported. A reactor design that has gained some attention is the continuous extraction of water from the reaction medium in a wiper‐blade evaporator. In 2012, BASF SE^47^ reported a continuous method to dehydrate a carbohydrate‐containing feedstock in the presence of an IL and an organic co‐solvent. The reaction solution was then evaporated and the HMF‐containing, gaseous discharge of 421.1 g h^−1^ was condensed and separated from the organic solvent. In the given examples, a continuous feed of [BMIm]Cl (300 g h^−1^) and a mixture of fructose/methanol/water (1:1:1) at 22.3 g h^−1^ are merged and evaporated at 200 °C to give an HMF yield of 8 %. Using 200 g h^−1^ 1‐hexyl‐3‐methylimidazolium chloride ([HMIm]Cl) feed and 44 g h^−1^ fructose/methanol/water at 170 °C, the yield increased to 10.1 %. A critical open question is what factor exactly influences the increase in yield, whether it is the change of the IL or the lower temperature and shorter reaction time in the evaporator, or a combination of those. The short reaction time in the evaporator, the implementation as continuous method and the abundance of a catalyst are stated as advantages of the method. However, these advantages cannot compensate the low yield of HMF, which makes optimization of the process necessary. In general, the large amounts of organic solvent mixed with ILs pose disposal problems that call for elaborated recycling of the reaction media, which in turn leads to more complex processes and higher production costs.

### 4.2 Archer Daniels Midland Company

In a patent assigned to Archer Daniels Midland Company (ADM) (Chicago, USA),^48^ several examples (see Table 3) are listed to illustrate the effect of temperature, solvent, and distillation on the HMF yield. The highest yield of 80.6 % at a conversion of 94.1 % was obtained in a batch reactor using *N*‐methylpyrrolidinone (NMP) as the solvent and the commercial ion‐exchange resin Amberlyst 35 WET as the catalyst at 115 °C for 300 min. A temperature reduction of 10 °C led to a reduction in HMF yield to 71.6 %. When changing the solvent to *N*,*N*‐dimethylacetamide (DMAc) at 105 °C under the same reaction conditions as above, the HMF yield dropped to only 62.1 %. When performing an in situ distillation at 105 °C for 120 min using NMP and Amberlyst 35, the HMF yield was 75.7 %. Purification processes for each example are given in the patent as well. Although there are many studies using ion‐exchange resins for the dehydration to HMF, there is a limitation to the applicable reaction temperature. Typically, temperatures below 150 °C are tolerable for these catalysts. The review of Qiao et al.12b showed that high HMF yields could be obtained with ion‐exchange resins and biphasic systems, organic solvents, and ILs. Another apparent drawback of this method is the use of hazardous solvents, such as DMAc and NMP.

ADM^49^ also developed a HMF production method employing microwave irradiation as heating source. The highest HMF yield was 77.7 % at a fructose conversion of 80.1 %. Further, 12.4 % of side‐products were formed. The synthesis was performed in NMP with 1.8 % H_2_SO_4_ at 160 °C for 20 min.

In another ADM^50^ process, the fructose‐containing feedstock, water, a homogeneous acid catalyst, and a solvent are added in a reactor and converted to a defined partial conversion endpoint that did not exceed 80 mol % of theoretical HMF yield. The reaction mixture is then quenched and neutralized. Separation and purification of HMF is done by liquid–liquid extraction, phase separation, and filtration of humins. The large amounts of organic solvents typically needed in liquid–liquid extraction processes makes them less sustainable. The maximum HMF yield reached in this process was 49 %, using HCl and 2‐butanol as the catalyst/solvent couple. The reaction was performed at 120 °C for 30 min. NaCl was also added to increase the partition coefficient of HMF in the biphasic water/organic solvent system. The salting‐out effect, induced by NaCl, resulted in an increased immiscibility of the aqueous and organic phase, which improved the extraction of HMF from the aqueous phase and consequently reduced unwanted side reactions in water. In the patent, ethyleneglycoldimethylether (glyme), 1,4‐dioxan, bis(2‐methoxyethyl)ether, and THF were also tested as solvents, but the HMF yield was even lower. This is in accordance with previous work by Román‐Leshkov and Dumesic,^51^ who extensively studied the impact of different salts on the HMF yield as well as the impact of the solvent in biphasic systems saturated with NaCl in laboratory experiments. They concluded that within the studied solvent classes (primary and secondary alcohols, ketones, and cyclic ethers in the C_3_–C_6_ range), C_4_ solvents gave the highest HMF yield. In addition, NaCl and KCl lead to the highest extraction power and HMF selectivity.

In a recent publication,^52^ it has been shown that hexafluoroisopropanol, a low‐boiling extraction solvent, has a partition coefficient superior to solvents such as MIBK or *n*‐butanol. The easy isolation of HMF, the good selectivity, and easy recyclability are clear advantages of this solvent. This highlights again the importance of proper solvent and catalyst selection.

ADM^53^ designed a process for HMF production, in which unreacted sugars are directly fermented to ethanol. High fructose corn syrup with 42 % fructose content (HFCS‐42), was used in the acid‐catalyzed dehydration. The HMF yield was set to about 20 %, which is too low to be of interest for an economic process.

### 4.3 Mikromidas Inc.

**Figure 5:**
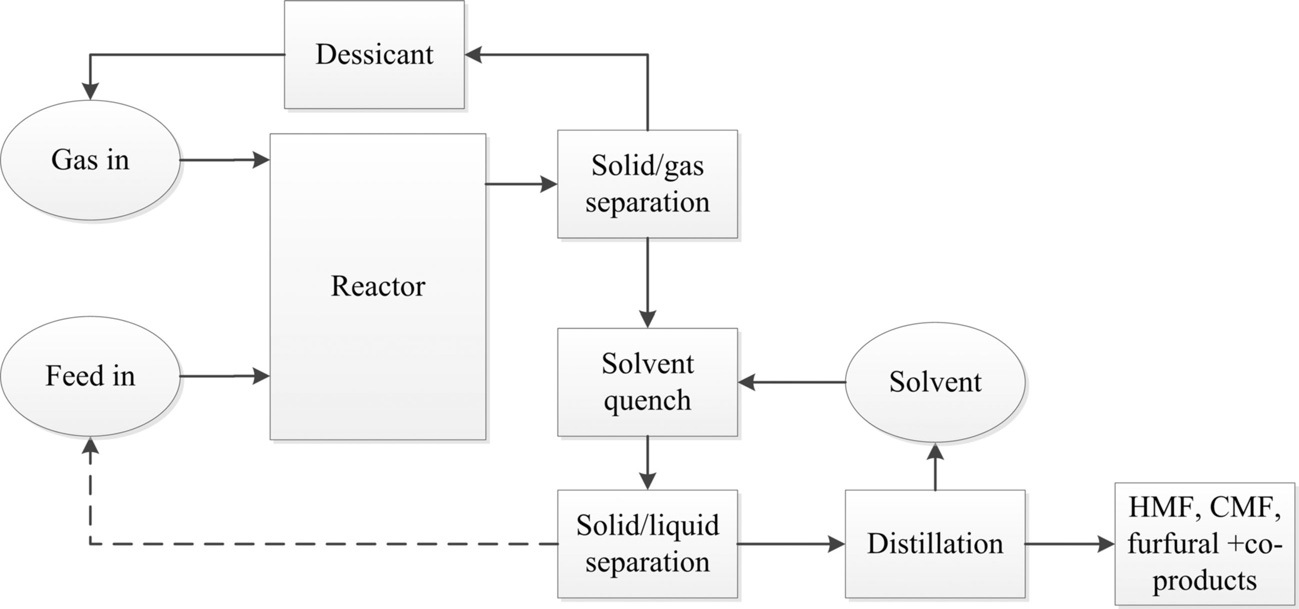
Multiphase reaction process for HMF production by Micromidas.^54^

A remarkable process technology with regard to the reactor type was disclosed by Micromidas Inc. (West Sacramento, USA).^54^ They described a process utilizing a multiphase reactor, for example, a fluidized bed reactor, for the conversion of cellulosic feedstock to chloromethylfurfural (CMF). The production of HMF was also mentioned. Biomass and dried gaseous acid, for example, HCl gas, is continuously fed into a multiphase reactor. The reaction is performed at temperatures between 200 °C and 250 °C. As can be seen in Figure 5, the separation of the gaseous acid and the reaction mixture is done by using a solid–gas separator, for example, a cyclone, a filter or a gravimetric system. Suitable solvents for the purification are, among others, dichloromethane or hexane. With regard to sustainability and commercial‐scale application, the use of a cellulosic feedstock is preferable. The advantages of performing the reaction in a fluidized bed reactor are the rapid mixing of the suspended solid around the bed, the uniform heat transfer and the elimination of hot spots within the reaction mixture. The mixing also reduces the need for pretreatment of the biomass. CMF and furfural are reported as main products. For the production of CMF, a total yield of 35 % was given. Unfortunately, no HMF yield was disclosed in the patent. The uniform mixing and temperature gradient are clear advantages of fluidized bed reactors. Kinetic studies17a, 17c indicate that the reaction time and temperature are critical parameters for the dehydration to HMF. The corrosiveness of HCl (g) is a serious problem of this system.

### 4.4 Wisconsin Alumni Research Foundation

Wisconsin Alumni Research Foundation (WARF) (Madison, USA)^55^ studied the production of HMF with and without catalysts from various carbohydrate feedstocks in DMAc/LiCl in 2009. The conversion of fructose without additional catalyst gave moderate yields of 55–65 % at temperatures of 80–140 °C. The yield was significantly improved by the use of Brønsted acids, for example, H_2_SO_4_, and Lewis acids, for example, CuCl_2_. The addition of ILs was also beneficial. The highest HMF yield (94 %) starting from fructose was obtained in a system using H_2_SO_4_ as catalyst and DMAc/LiCI as the solvent at 120 °C and 12 min reaction time. A problem that has been overlooked is the limited stability of DMAc at elevated temperatures, especially in the presence of acidic catalysts, which causes hydrolysis and formation of dehydracetic acid‐type condensation products.^56^

In addition, DMAc is classified as “hazardous” according to the Chem21 Initiative.^28^

**Figure 6:**
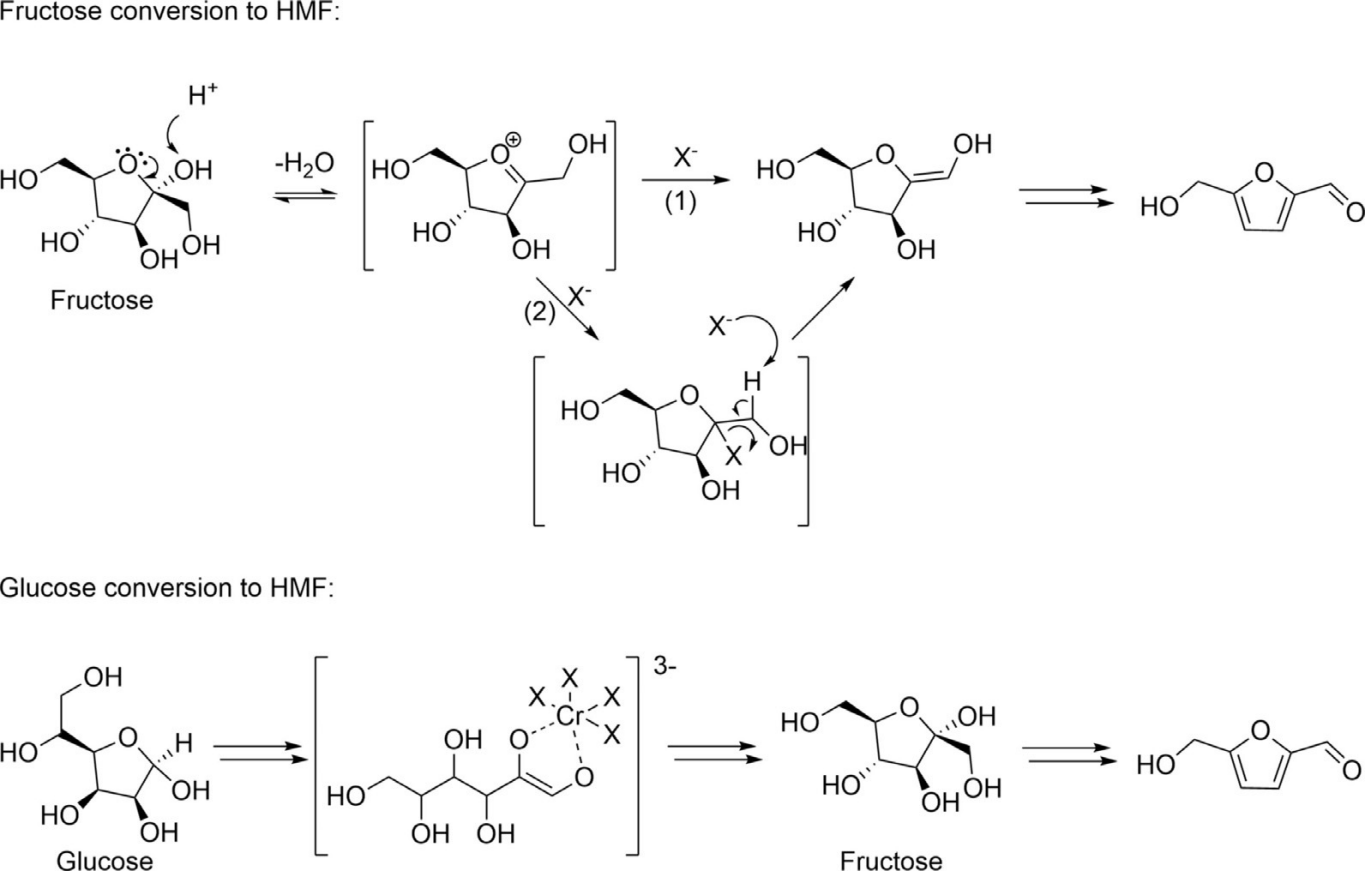
Influence of metal halides on mechanism of HMF formation from fructose, (1) base pathway and (2) nucleophilic pathway (top), and influence of metal halides on glucose conversion (bottom) as proposed by WARF^[55]^.

In general, the proposed cyclic pathway of HMF formation involves the formation of a fructofuranosyl oxocarbenium ion, which is then deprotonated at C1 to form an enol and subsequently an aldehyde (see Figures 2 and 6). Based on their experimental data, WARF proposed two variations of the fructose conversion mechanism, taking the effect of metal halides into account (see Figure 6).

In the base pathway, the halide ion (X^−^) deprotonates the C1 and forms the enol. This pathway must be considered highly unlikely due to the low basicity of chloride. In the nucleophilic pathway, the halide ion forms a 2‐deoxy‐2‐halo intermediate that was proposed to be more stable and less prone to side reactions. Upon HX elimination, the enol is formed. By adding ILs, for example, [EMIm]Br, a higher HMF yield of 94 % was achieved (see Table 3). The conversion of glucose to HMF was studied as well. The highest yield of 81 % was obtained using CrCl_2_ and NaBr as the catalyst and DMAc as the solvent at 100 °C for 300 min. The results of the experiments indicated that bromide was the most effective halide ligand for the conversion of glucose. It was also stated that halides served as ligands for the chromium atom and influenced the selectivity of the reaction.

The HMF yields from the conversion of cellulose (54 %) and mannose (69 %) were comparably lower. Moreover, although the described experiments have provided mechanistic insights into carbohydrate conversion in DMAc, the approach has weaknesses also with regard to the low amounts of reaction solution used in addition to the above‐mentioned instability of DMAc under the applied conditions. The experiments have reportedly been done in small glass vials, leaving much doubt whether a successful scale‐up could be possible.

In 2016, WARF^57^ patented a process for HMF generation from cellulose in polar aprotic solvents, for example, THF, in the absence of water. Cellulose is first degraded to levoglucosan and then dehydrated to HMF. The highest HMF yield was 44 %. A comparison of the described processes is given in Table 3. The utilization of a low‐boiling‐point aprotic polar solvent facilitates the separation of HMF. Compared to the previously described cellulose conversion, the HMF yield is rather low. This might be attributable to the fact that levoglucosan formation from cellulose generally needs higher temperatures of about 180 °C. In these experiments, 60 mL reaction solution was used for the conversion in a batch reactor.

Besides monophasic systems, biphasic water–solvent systems were studied. Monophasic systems have a major drawback regarding the separation of the catalyst from the solvent. This can be overcome by biphasic systems, in which the catalyst remains in the water or IL phase and HMF is transferred into the organic solvent. This also reduces the risk of rehydration of HMF to levulinic acid and formic acid. There have been numerous studies on biphasic systems described in literature for lab‐scale synthesis of HMF.11a

Besides the previously described process by ADM,^50^ some major contributions regarding biphasic systems for the development of industrial scale processes came from WARF^58^ Biphasic reaction systems in the presence of an acid catalyst and a chemical modifier were studied, the latter comprising an inorganic salt, for example, metal halides, and a dipolar aprotic additive. Kazi et al.^59^ published a techno‐economic analysis of the process. In this theoretical analysis fructose was used as feedstock and water and butanol as solvents in the biphasic reactor. HCl and NaCl are used as the catalysts. The annual capacity of the hypothetic HMF production was set to 61 000 metric tons, operating with 300 metric tons of fructose per day. Kazi et al.^59^ calculated the investment costs for a plant with a HMF production yield of 61 kt per year and a lifetime of 20 years at approximately 110 873 000 €* (*at a currency rate of 0.7 €/$ [10.11.2010]). This value is based on HMF production investment of 1 302 000 €*, HMF separation of ≈24 759 000 €*, and fructose and levulinic acid recovery of 45 598 000 €* as equipment costs and ≈39 214 000 €* for additional costs (e.g., engineering, legal expenses, etc.). Operational costs were calculated with ≈45 668 000 €* per year including 22 064 000 €* per year for feedstock. The minimum selling price of HMF was approximately 0.9 €* L^−1^. They concluded that a better performance of the process, for example, by increasing the yield, is necessary to overcome economic uncertainties.

In 2014, WARF^60^ developed a similar method for glucose conversion to HMF in a biphasic reactor. The dehydration of glucose was performed using a homogeneous Brønsted acid, such as mineral acids, and Lewis‐acidic metal halides, such as AlCl_3_, SnCl_4_, VCl_3_, InCl_3_, GaCl_3_, LaCl_3_, DyCl_3_, or YbCl_3_. The yield of HMF was 62 % using AlCl_3_ and HCl as the catalysts and *sec*‐butylphenol (SBP) as the organic extraction solvent. The utilization of expensive solvents such as DMAc is avoided. In addition, the separation and purification of previous processes was cost and time consuming. The low yield compared to previous processes from WARF was a significant drawback for industrial‐scale application. In a similar method,^61^ various lactones, furans, and pyrans were used as organic extraction solvents for the conversion to HMF. Systems using γ‐valerolactone (GVL), 5‐butyloxolan‐2‐one (GOL), 5‐propyloxolan‐2‐one (GHL), and 5‐heptyloxolan‐2‐one (GUL) were given in the examples. Reacting fructose to HMF in a biphasic system with GVL gave a conversion of 94 % and a selectivity of 84 %. The existing process technologies with biphasic systems still face some challenges regarding low HMF yield that need to be overcome for an economic large‐scale production. As can be seen in Figure 1, GVL can be derived from HMF or more precisely from levulinic acid. GVL is classified as “problematic” according to the Chem21 Initiative.^62^

### 4.5 Battelle Memorial Institute

Not only WARF^55^ demonstrated that the addition of ILs to the reaction medium increased the HMF yield. Other patents by Battelle Memorial Institute (Columbus, USA)^63^ also indicated such improvement. They described a method for the conversion of fructose to HMF using ILs and metal halide catalysts, such as CrCl_2_. The formation of side products, such as levulinic acid, formic acid, or humins, were major drawbacks when performing the HMF synthesis in aqueous solutions. This problem was overcome by using ILs, leading to higher conversion rates and HMF yields. Experiments were performed with fructose and glucose at 80 °C. An HMF yield of 63–83 % was obtained from the conversion of fructose and 68–70 % when glucose was used as the feedstock. Previous studies by WARF^55^ demonstrated that a significantly higher HMF yield for fructose (81–94 %) and glucose conversion (78–81 %) is possible when using DMAc and metal halides. The influence of metal halides as catalyst on the glucose conversion is schematically given in Figure 6 (bottom). A major drawback of the utilization of ILs is typically the costly recycling of the solvent. Battelle Memorial Institute63b also developed an adsorption separation process for ILs, describing a method and an apparatus for the separation of reaction products, such as HMF, from ILs, thus providing a way to reuse the costly IL medium.

### 4.6 Agency for Science, Technology, and Research

**Figure 7:**
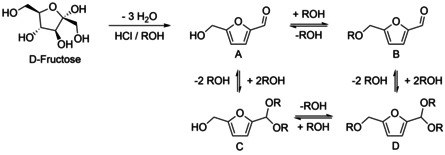
Conversion of fructose to HMF (product A) and its derivatives (products B–D) in alcoholic solvents (R=methyl, ethyl).^65^

HMF yields comparable to the ones from Battelle Memorial Institute were obtained by the Agency for Science, Technology, and Research (A*STAR, Singapore), which developed^64^ a process for the dehydration of carbohydrates to HMF in alcoholic solvents. The highest HMF yield of 83 % obtained using isopropanol and HCl as catalyst at a temperature of 100 °C and a reaction time of 4 h. Ethanol, 1‐propanol, and 1‐butanol were tested as solvents as well, but the HMF yield was reduced due to ether formation (see Table 3). In the work up procedure, NaOH was added for neutralization and the solvent was removed by vacuum distillation. Then, the product was dissolved in water and extracted with ethyl acetate, which in turn was removed by distillation to give the crude product. Isopropanol is a very good solvent regarding sustainable processing, it is classified as “recommended” according to the CHEM21 solvent‐selection guidelines. Lai and Zhang, the inventors of the patent, also discussed their finding of the fructose conversion in isopropanol in a publication.^65^ The main by‐product in the conversion of fructose in isopropanol was high‐boiling humins, which were removed by filtration. In addition to the formation of the target product A, the formation of by‐products B–D (see Figure 7) was reported for methanol as the solvent. In ethanol, the main products formed were A (HMF) and by‐product B, and in isopropanol and *tert*‐butanol mainly HMF was formed. In this publication, also the influence of different Brønsted acids was addressed; the highest yield was reported for HCl, followed by H_2_SO_4_. HNO_3_, H_3_PO4, HCOOH, CH_3_COOH, and B(OH)_3_ were also tested but gave only traces of HMF or no HMF at all in isopropanol. Amberlyst 15 was tested as a solid‐acid catalyst in different alcohols and caused increased etherification and acetalization.

The conversion of carbohydrates to HMF in alcoholic solvents should certainly be further developed. In addition, the suppression of humin formation must be a central topic to be explored in future research.

### 4.7 Sartec Corporation

Another HMF‐generation approach using alcohols as solvents was developed by Sartec Corp. (Anoka, USA)^66^ In this process, a saccharide solution is in contact with a metal oxide catalyst (TiO_2_) at temperatures between 180–200 °C. The single‐step reaction is continuously performed in a column reactor packed with TiO_2_ particles. Monophasic solvent systems (isopropanol, ethanol, methanol) were tested in the conversion of saccharose using ZrO_2_ or TiO_2_ and gave rather low HMF yields of 10–14 %. Biphasic solvent systems (butanol/water or MIBK/water) were also used for the conversion of glucose, fructose, and saccharose. The highest HMF yield was 46 % for the conversion of glucose in MIBK/water (10:1) at 180 °C for 2 min in a TiO_2_‐packed column. HCl was used as a co‐catalyst. No further work‐up procedure was given in the patent; the yields were analyzed by HPLC from the organic and aqueous phase. Interestingly, the overall HMF yield was rather low in butanol/water systems (<20 %). Previous studies by Román‐Leshkov and Dumesic^51^ revealed that the HMF selectivity of 1‐butanol systems is rather low, especially compared to 2‐butanol. In the Sartec Corp. process, HMF was detected in both the organic and the aqueous phase in considerable amounts. This shows that the described method has some apparent problems with the extraction efficiency of the organic phase. It has been shown in previous studies^50^, ^51^ that the extraction sufficiency can be improved by the addition of inorganic salts (see above). In the process by Sartec Corp., this was not considered. Another apparent limitation is the lack of information on possible work‐up procedures. Even though the continuous production in a packed column reactor is an interesting approach, evidently further developments and improvements of the method are needed.

### 4.8 Novamont S.P.A.

**Figure 8:**
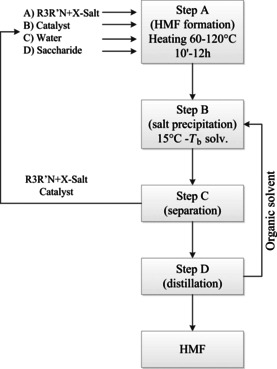
HMF process proposed by Novamont S.P.A.67a

Besides the solvent system, also the used catalyst obviously impacts the HMF yield. Patents assigned to Novamont S.P.A. (Novara, Italy)^67^ disclose a dehydration process of saccharides to HMF using various catalytic systems. The flowchart of the process is given in Figure 8. The reaction mixture consists of a catalyst (either TiO_2_ supported on immobilized SiO_2_, phosphotungstic acid supported on SiO_2_ (HPWO/Si50O), α‐Zr(HPO_4_)_2_ or Ti(HPO_4_)_2_, a quaternary ammonium salt, water, and a saccharide as feedstock. The quaternary ammonium salt can be tetramethylammonium chloride (TMAC), tetraethylammonium chloride (TEAC), tetraethylammonium bromide (TEAB), or tetrabutylammonium bromide (TBAB). An organic solvent is used for the extraction of HMF from the aqueous reaction mixture. In the given examples, saccharose was dehydrated at 80 °C for 15 min and then the temperature was increased to 100 °C. TEAB and HPWO/Si50O as catalyst and 2‐butanone as extraction solvent were used. HPWO/ Si50O was synthesized from H_3_PW_12_O_40_ and commercial SiO_2_. The HMF yield was 67 % and the purity 94.1 %. When this reaction was performed with fructose, the yield was 80 % at 99.6 % purity. In another example, fructose was dehydrated in the presence of TiSi50O as catalyst and TEAC as salt. The synthesis of the TiSi50O catalyst from dioxane, SiO_2_ and titanium isopropoxid [Ti(*i*PrO)_4_] was also described in the patent. Ethanol, chloroform, and THF were used for the separation and purification of HMF. The yield was 93 % and the purity of HMF was 97.6 %. The solid catalysts were recycled afterwards. After the third cycle, the HMF yield dropped to 82 %. When using α‐Zr(HPO_4_)_2_ and TEAB as catalyst, 87.6 % HMF yield with 99 % purity was obtained. The good HMF yields as well as the relatively low reaction temperatures are advantages of the method. The applicability of these results to large quantities is certainly an interesting topic for future research. However, the use of toxic and water‐contaminating quaternary ammonium salts should be avoided.

### 4.9 Novozymes A/S

Several patents assigned to Novozymes A/S (Bagsværd, Denmark)^68^ disclosed a continuous method for the salt‐catalyzed dehydration of fructose to HMF. The reaction medium consists of an organic phase and an aqueous phase containing the salt. Besides fructose conversion, also the utilization of mannose or glucose is stated in the patents. The enzyme glucose isomerase converts glucose to fructose, and correspondingly mannose isomerase converts mannose to fructose. The highest HMF yield obtained was 70 % at a fructose conversion of 98 % and a selectivity of 72 %. KCl was used as a salt catalyst in this experiment. These findings clearly indicate that the selection of a proper catalyst is essential. The glucose isomerase enzyme showed good stability in the presence of NaCl, KCl, and Na_2_SO_4_. A number of interesting research questions, for example, the effect of HMF and salt on the activity of the isomerase enzyme and the conversion of glucose, fructose, and mixtures of both in MIBK/water systems were investigated. Unfortunately, a complete process starting from monosaccharide to HMF using isomerase enzymes was not included in the patent, leaving the question of the scalability of this system open.

### 4.10 UC Regents

Previous studies by Novozymes A/S did not discuss the applicability of a complete process using isomerase enzymes. Research by UC Regents (Oakland, USA) investigated this issue further. A process in which glucose is enzymatically converted to fructose was assigned to UC Regents^69^ in a patent on the production of HMF in ILs, for example, EMIm‐based ILs. Glucose was used as a starting material and was enzymatically converted to fructose with glucose isomerase and borate salts. An acid catalyst, for example, AlCl_3_, was used for the dehydration of the fructose to HMF, the highest yield of HMF in these conversion examples was in the range of 55–60 %, which is rather low compared to other processes. Another example of the conversion of cellulose to glucose in [C_4_mim]Cl using HCl at 140 °C for 60 min was included. In the given example, glucose was treated with glucose isomerase and sodium borate to yield fructose, which was then reacted in [C_2_mim]Cl for 30 min at 100 °C to yield HMF. Data on the isomerization efficiency is missing, it would be interesting to compare the turnover frequency and the total turnover number of the described enzymes to those of industrial isomerization processes.

### 4.11 Südzucker AG

The production of HMF in aqueous systems faces major challenges due to the formation of side‐products, especially when mineral acids are used as catalysts. The main challenge in the continuous production of HMF in aqueous systems are the solid side‐products.

**Figure 9:**
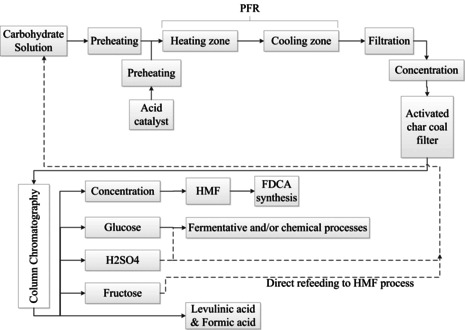
Continuous process for HMF production by Südzucker AG.70a

The setting of a partial conversion endpoint is an interesting method to increase the HMF yield and to reduce the formation of side‐products. A continuous process that uses this method was patented by Südzucker AG (Mannheim, Germany).^70^ The Südzucker process is given in Figure 9. Mineral acids, such as HCl, H_2_SO_4_, or H_3_PO_4_ (1–2.5 wt %), were used for the dehydration of fructose to HMF in aqueous media at temperatures in the range of 80–165 °C in a plug flow reactor (PFR). The maximum conversion of fructose was 40 %, HMF was purified by column chromatography. For a sustainable process, all produced streams must be valorized and resupplied to the process. Due to the low HMF yield, additional process steps, for example, additional purification steps to isolate the unreacted carbohydrates, are needed, which also affects the cost and efficiency of the process.

Previous studies also investigated this HMF formation setup (aqueous system, fructose as feedstock, mineral acids as catalyst) in continuous processes. Many of the described continuous systems applied a microreactor system due to the better mixing and heat transfer. Tuercke et al.^71^ used 10 % fructose solution mixed with 0.1 mol L^−1^ HCl (1:1) to obtain 54 % HMF yield in a microreactor. Higher fructose concentrations were only used when organic solvents were added to the system. 60 % HMF yield was obtained by Muranaka et al. ^[72]^ (5 min at 180 °C in a microreactor). Very low fructose concentrations of 1 wt % in phosphate‐buffered saline were used. The monophasic experiments showed an increase in side‐products compared to biphasic systems.

Consequently, the continuous production in a microreactor is also limited by the formation of solid side‐products. Typically, the reactions are performed at very low fructose contents to minimize humins formation and reduce the risk of clogging.

### 4.12 AVALON Industries AG

**Figure 10:**
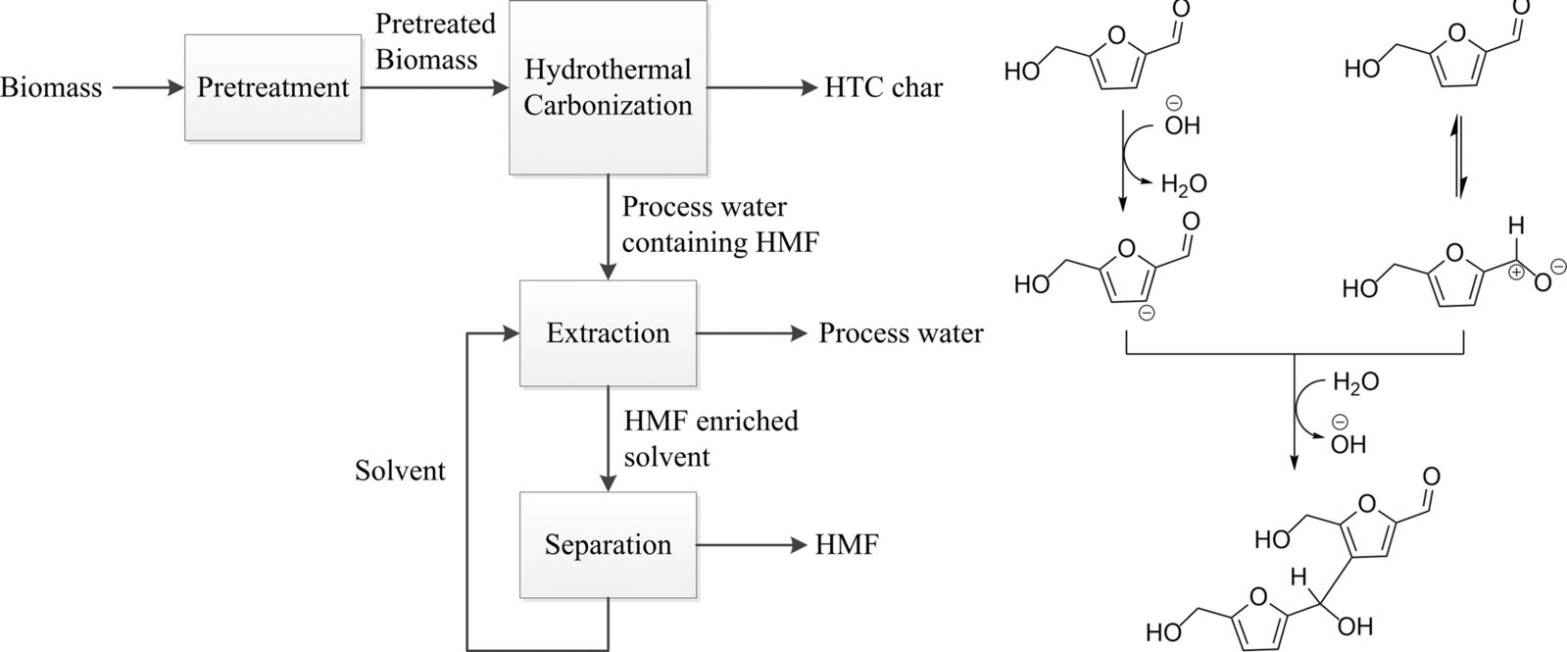
Hydrothermal process by AVA Biochem (left)^73^ and proposed HMF oligomer formation (right).75b

The previously described processes focused on the conversion of mono‐ or disaccharides to HMF. AVALON Industries AG (Zug, Switzerland)^73^ developed a process for the conversion of lignocellulose to HMF. They filed several patents on the hydrothermal carbonization (HTC) of lignocellulose to produce HTC char, which can be used as energy source.^74^ In this process, lignocellulose is decomposed under high temperature and pressure to glucose and then fructose, which is dehydrated to HMF. Utilization of fructose as feedstock would enhance the formation of HTC char and reduce the HMF yield. The lignin from the lignocellulose slows down this formation and ensures a constant dehydration of fructose to HMF. The utilization of a lignocellulosic feedstock is a clear advantage of this process. The HMF‐containing process water is then extracted with a solvent, for example, supercritical CO_2_, in a countercurrent mix‐settler column process. The HMF‐enriched solvent is then subjected to another separation step. In case of supercritical CO_2_, a dedusting technology is used for the separation of HMF and the supercritical CO_2_. The company also issued patents^75^ on carbon‐linked HMF oligomers that contain at least two HMF units. The proposed structure is given in Figure 10. The main advantage of this HTC process is that HMF is produced as a side‐product that is dissolved in the process water. This generates additional revenues. Unfortunately, no HMF yield is given to evaluate the efficiency in more detail. In contrast to the previously described processes, the HTC technology has already been scaled up to small‐scale commercial production of HMF.

An overview of relevant factors of the described processes is given in Table 3.

The conversion of cheap lignocellulosic materials to HMF is a highly relevant topic, especially with regard to a production at larger scale. The industrialization of many of the described processes is hindered by the formation of hydrochar (humins that are polymerized in different ways than the humins formed by the acid‐catalyzed process). The structure depends on many parameters, for example, used feedstock and synthesis parameters. Many models have been proposed for the hydrochar formation from hydrothermal carbonization of carbohydrates. Patil and Lund^76^ suggested that the initial step in the hydrochar formation is the hydrolytic ring opening of HMF and the formation of aldol condensation products, such as 2,5‐dioxo‐6‐hydroxy‐hexanal (DHH). Shi et al.^77^ proposed that α‐carbonyl aldehydes, such as DHH, pyruvaldehyde, and 3‐deoxyglucosone are key primary precursors for the formation of hydrochars.

## 5 Commercial HMF Production

Since 2014, AVA Biochem produces HMF at a commercial small scale. AVA Biochem is a subsidiary of AVALON Industries AG, which announced that they will focus on the global implementation of the HTC technology for industrial‐scale HMF production.^78^ Even though the AVA Biochem plant claims to have reached technology readiness level (TRL) 9, corresponding to full commercial application with the developed system proven in operational environment,^79^ it is still not operating a large‐scale, industrial production—the operating capacity of the plant in Muttenz, Switzerland, being 300 t per year. In 2019, AVA Biochem announced that it is currently planning the next scale up to 5000–10 000 t per year.^80^ In October 2018, an on‐farm biorefinery technology center opened at Stuttgart‐Hohenheim in Germany, the core of the small‐scale plant being an HMF module. In the EU project “Grace” (2017–2022), Miscanthus biomass is used as starting material.^81^

Up to present, no commercial, large‐scale HMF plants are running, which is reflected in the still rather high price of HMF (Sigma–Aldrich, 3500 € kg^−1^).

## 6 Analysis of Issues of Scaled Production

Several processes for the production of HMF have been developed and patented by companies.

AVA Biochem already set the first steps toward industrial production with a small‐scale operating plant. Many companies contributed to the development in patenting various process technologies, but there are still many problems that have to be dealt with. The main challenges in the upscaled HMF production are the formation of side‐products, especially of solid humins, the separation of HMF from the reaction media, and its subsequent purification. In general, by‐product formation depends on the reaction parameters and affects the purification and ultimately also the economic efficiency of the HMF production.

Many of the described processes are still at a very early stage of development, in which media and parameter optimization is the main focus.

### 6.1 Formation of side‐products

The increase of efficiency and reduction of side reactions is essential for an economic and sustainable process. In general, it is better to prevent the formation of side‐products from the beginning unless they can be valorized. Formation of levulinic acid and formic acid does reduce the HMF yield, but this presents only a minor challenge because of their usability.

The formation of condensation products—humins—is much more problematic. Recently, the valorization of humins has come into focus as they are considered a key factor for an economically feasible process. This valorization of humins also presents several challenges, as their chemical structure and yield is process dependent and their separation rather demanding.

Many publications focused on the analysis of HTC humins, which are also referred to as hydrochar. Unlike the acid‐catalyzed dehydration of carbohydrates, the HTC process does not involved acids. Consequently, the resulting structure of the HTC humins is different from the humins produced according to acid‐catalyzed dehydration.

The molecular structure of hydrochar as well as its formation kinetics are still under debate. Shi et al.^77^ summarized a formation route for hydrochar as follows:

1.: Biomass (cellulose and hemicellulose) are hydrolyzed to monosaccharides.
2.: Monosaccharides are dehydrated to furanoic compounds, for example, HMF.
3.: The formed compounds undergo a series of polymerization–polycondensation reactions leading to the formation of polyfuranic compounds.
4.: These polymers further undergo aromatization to form a polyaromatic hydrochar structure.

Humins are still mainly used for energy or heat generation, even though higher value‐added applications are desired. Hoang et al.^82^ analyzed the application of humins in gasification or steam‐reforming processes for hydrogen production. The pyrolysis of humins for the production of liquid fuels and biochar is described in the literature as well.^83^ The pyrolysis of humins led to a wide range of compounds, the primary volatile compounds being furans and phenols. An increased pyrolysis temperature caused aromatization of the products. Further research is still required to understand mechanism and kinetics of humin pyrolysis.

The potential of humins in higher‐value applications, such as thermoset materials, was investigated by Mija et al.24a as well as Sangregorio et al.24b Tosi et al.^84^ reported the production of macroporous foams from humins. Its application as feedstock for the production of activated carbons and the utilization as potential electrode materials for supercapacitor applications was also studied.^85^

The valorization of side products—humins in our specific case—is one approach to make a process more efficient. Nevertheless, humins formation might reduce the HMF yield drastically.

### 6.2 Separation and purification of HMF

Many earlier publications dealt with the prevention of condensation reactions since they cause major problems in HMF separation and purification. An increasing number of publications focus on the kinetics of the dehydration to HMF. Dashtban et al.^4^ reviewed the kinetic analysis of HMF for aqueous, biphasic and IL systems.

There have been numerous studies on the fructose conversion to HMF in polar aprotic co‐solvents, such as DMSO. Typically, high HMF yields and fewer by‐products are formed in systems using DMSO as solvent. This has been also shown in the process developed by Roquette Freres.^38^ Some aspects of the role of DMSO in the conversion to HMF are still debated, for example, the ability to increase the reaction rate in the absence of an acid catalyst. Recently, Whitaker et al.^39^ showed that the fructose conversion in DMSO is mainly promoted by solvation effects and does not originate from H_2_SO_4_ catalysis. This is in line with general knowledge from synthetic carbohydrate chemistry that often uses DMSO as advantageous solvent for water elimination reactions from furanoid and pyranoid systems. So far the following roles of DMSO in the fructose conversion have been proposed:^39^, ^41^, ^43^

1.: DMSO alters the isomer distribution of fructose.
2.: DMSO promotes conversion through solvation effects.
3.: DMSO reduces HMF susceptibility to nucleophilic attack.
4.: DMSO stabilizes HMF in solution.

Even though the formation of side‐products is significantly reduced in DMSO and other solvents with high boiling points (*T*_b_), the recovery of HMF from these solvents is a major issue in large‐scale production. Typically, the purification processes involve distillation or extraction, for example, with ethyl acetate. Depending on the boiling point of the solvent and its affinity to HMF, these separation and purification processes can be very cost intensive and might require large amounts of organic solvents.

The same holds true for biphasic systems containing organic solvents and water. It needs to be critically mentioned that literature accounts often do not describe purification or separation processes. In many cases the HMF yield is determined by HPLC, such as in a process^66^ using MIBK/water as the biphasic reaction system.

Processes using alcohols as solvents, have been described as well. The HMF yield in alcoholic solvents is reduced due to side reactions, such as ether formation and acetalization. In general, alcohols are favorable solvents since they are environmentally friendly and give good HMF yields. Separation from alcoholic reaction media can be done by low temperature vacuum distillation as has been shown by A*STAR. The easy separation is a clear advantage of alcoholic and low‐boiling point systems.

From an environmental perspective, water would also be a good solvent, but the pronounced formation of side products is a major issue here. The continuous production of HMF in aqueous systems was reported as well but was impeded by the formation of solid side‐products, which remains a general problem in the continuous production of HMF in aqueous systems catalyzed by mineral acids.

### 6.3 Catalyst regeneration

Catalyst separation and recycling are additional aspects to be considered for a sustainable process. The separation of a solid catalyst from the reaction media can be done quite easily by filtration. More challenging is the separation of catalyst from solid by‐products and the risk of catalyst inactivation. Novamont S.P.A^67^ described the production, separation, and recycling of various catalysts as well as their performance after reuse. They reported a drop in HMF yield after several cycles, although they were still rather high (82 %).

### 6.4 Economic considerations

Most of the described processes are still in a very early development stage, in which media and parameter optimization are in focus. The influence of solvent, catalyst, and feedstock on HMF yield and selectivity were investigated.

Many publications focus on the use of monosaccharides, especially fructose, as the starting materials for the conversion reaction (see Table 3). From an economic point of view, lignocellulosic biomass is much cheaper than neat fructose. The HMF production process developed by AVA Biochem^73^ is based on hydrothermal carbonization of lignocellulosic feedstocks. As it is one of the few implemented small‐scale processes, it can be assumed that the HMF yield is sufficient for an economic process. Although for the utilization of HMF as building block in the chemical industry, a significant lower price must be targeted.^39^, ^82^, ^83^, ^84^, ^85^

Evaluation of economic feasibility and the likelihood of the implementation of a process design at larger scale are important future research topics. Only a few techno‐economic analyses of HMF production processes have been published so far. A techno‐economic analysis of a process developed by WARF^59^ gave a rather high selling price of HMF. It was concluded that a better performance of the process, that is, a higher HMF yield, is needed to bring about guaranteed economic feasibility. For an economic feasibility, sufficient material must be available for an acceptable price. As a reference, the production of particle boards alone requires some 5–7 million tons of low priced standard adhesive each year.^86^ Further techno‐economic analysis on HMF production processes are necessary for a better comparison of the economic feasibility of different technological options. In a recent publication, Motagamwala et al.^87^ reported a process for the production of HMF from fructose using acetone/water as solvent and HCl as catalyst. They obtained 89 % HMF yield with an HMF selectivity of 95 %, after evaporation of the solvent under reduced pressure HMF was recovered with 99 % purity. They also developed a process model and performed a techno‐economic analysis of the process. They obtained a minimum selling price of HMF of 1567.2 €* (*at a currency rate of 0.9 €/$ [12.04.2020]), assuming a fructose cost of 745.7 €.

## 7 Summary and Outlook

There has been a continuous growth of interest in large‐scale HMF production. Several processes were described in literature, but they still face several challenges that need to be overcome beforehand. Several aspects ranging from the choice of starting material to the recycling of solvent and catalyst to the formation of side‐products need to be considered for a comprehensive process assessment. Many processes just target a specific problem instead of focusing on all challenges. HMF yield and reaction mass efficiency (RME) are good indicators of the efficiency of a process.

A method that addresses these challenges was obtained by Novamont S.P.A^67^ using TiO_2_ supported on SiO_2_ and a quaternary ammonium chloride salt (TEAC). This process has the highest HMF yield of 93 %. The reaction was performed in water. This highlights the importance of catalytic systems. As is the case in many of the described processes only very small reaction volumes were analyzed, leaving the open question of applicability of the process for larger‐scale production. The RME of this process was only 65.1 %. BASF SE^46^ described a semi‐batch system using ionic liquids (ILs) and obtained 86.5 %, which is the third largest HMF yield of the described processes (see Table 3). The RME of this reaction was 60.7 %. The high conversion rate of 97.6 % of this process indicates that the formation of follow‐up products was problematic as well but was significantly reduced by the choice of solvent system. No catalyst was used in this process. The positive effect of ILs on the HMF yield are in line with previous findings. The utilization of ILs is rather expensive, especially for large‐scale production, making solvent recycling essential. In addition, the isolation of HMF from ILs is rather challenging. A limitation of the BASF SE process is that only low fructose concentrations (20 wt %) were used and that the HMF yield dropped to 50.9 % with 65 wt % fructose solutions. This lowers the efficiency of the process dramatically.

Many of the described processes were performed in small glass vials. As solvent and catalyst recycling are important for the efficiency of a process, conversions in larger volumes are urgently needed and should be considered in future research.

Biphasic systems of organic solvent/water have a potential for scale‐up, but a high extraction efficiency and high solvent recycling rate are absolutely necessary for an economic production. The lack of data available for calculating green metrics that take the amount of waste into account is an aspect that should be considered in future research.

As can be seen in Table 3, most of the examples given in the patent literature were done with fructose as feedstock. When comparing the results obtained with fructose to those of other feedstocks, it must be pointed out that the HMF yield drops significantly when other carbohydrates such as glucose are used. The process developed by AVA Biochem uses lignocellulosic materials as feedstock.

## 8 Conclusions

Hydroxymethylfurfural (HMF) is a promising platform chemical for value‐added chemicals. It has a wide range of potential applications. Its relative instability, the degradation at higher temperatures and the formation of hard‐to‐separate side‐products are main challenges that need to be overcome for an efficient industrial HMF production process.

Taking the expected rising demand of HMF in the future into account, further improvements must be made to achieve a large‐scale operation.

## Conflict of interest

*The authors declare no conflict of interest*.

## Biographical Information

Catherine Thoma received her master's degree in Material Science from Technical University Vienna in 2018. She now works as a junior researcher at Kompetenzzentrum Holz GmbH in the area Wood Material Technologies. She is pursuing a PhD at the University of Natural Resources and Life Sciences. Her research interests involve carbohydrate conversion and sustainable production of carbohydrate‐based resins for wood‐based panels.